# Controversies in Urate-Lowering Therapy for Gout: A Comprehensive Review

**DOI:** 10.3390/gucdd3040019

**Published:** 2025-10-01

**Authors:** Michael Toprover, Michael H. Pillinger

**Affiliations:** 1 Division of Rheumatology, Department of Medicine, New York University Grossman School of Medicine, New York, NY 10016, USA; 2 Rheumatology Section, VA NY Harbor Health Care System New York Campus, United States Department of Veterans Affairs, New York, NY 10010, USA

**Keywords:** gout, urate-lowering therapy, colchicine, allopurinol, febuxostat

## Abstract

Gout is the most common inflammatory arthritis. Treatment of gout includes anti-inflammatory and urate-lowering agents. Robust guidelines by the American College of Rheumatology, the European Alliance of Associations for Rheumatology and other committees have been released regarding recommendations for urate-lowering therapy, including suggested first- and second-line medications, length of therapy with prophylaxis, and target serum urate concentration to treat patients to. Notably, the American College of Physicians guidelines do not recommend robust urate lowering and are more geared towards treating symptoms without monitoring or lowering urate. Controversies regarding the optimal management of gout patients still exist. In the following, we discuss several of these controversies and some of the most recent literature regarding potential future changes in recommended management of gout. We discuss options for prophylactic therapy and length, treating gout concomitantly with its most common comorbidities, hypertension, diabetes mellitus, and cardiovascular disease, potential debulking therapy for more severe gout, and optimal urate levels.

## 1. Introduction

The most common form of inflammatory arthritis, gout, is preceded by hyperuricemia, culminating in the deposition of monosodium urate (MSU) crystals and an inflammatory response to those crystals [1]. The treatment of gout requires a combination of anti-inflammatory therapies to manage and prevent gout flares, and urate-lowering therapy (ULT) to bring down systemic urate concentrations such that crystals cease to form and deposited crystals dissolve, in order to prevent future gout flares [2]. Despite advancements made in both treatment and guidelines, controversies remain about the optimal management of gout, and evolving data may challenge current management guidelines and lead to future changes in the treatment of gout patients.

Currently, some controversy prevails regarding (1) flare prophylaxis when initiating ULT, (2) the initial choice of ULT, (3) the efficacy and safety of currently approved agents, (4) target serum urate concentrations, and whether they may need to be more individualized. In this review, we will examine each of these controversies and the available data on the risks and benefits of each option.

## 2. Optimal Prophylaxis When Initiating ULT

The current gout management guidelines of the American College of Rheumatology (ACR) and the European Alliance of Associations for Rheumatology (EULAR) strongly recommend initiating prophylactic therapy with either colchicine or non-steroidal anti-inflammatory drugs (NSAIDs) over no prophylaxis, as well as continuing prophylaxis for a period of 3–6 months, rather than <3 months [2]. However, a newer study looking at whether prophylaxis was necessary by Stamp and colleagues interrogated these recommendations [3]. In a double-blind, placebo-controlled, non-inferiority study of 200 adults with gout who had at least one flare in the prior 6 months and met ACR criteria to start ULT, participants were started on allopurinol and randomized in a 1:1 fashion to either start 0.5 mg colchicine daily or placebo. After 6 months, ULT was continued but prophylaxis (colchicine or placebo) was discontinued. Using a prespecified non-inferiority margin of 0.12 gout flares per month, the authors found that, during the first 6 months, the colchicine group had 0.35 gout flares per month compared with 0.61 flares per month in the placebo group. However, by 12 months there was no difference in the mean number of gout flares per month between the two groups, with a *p* value of 0.68. At the same time, there were 11 serious adverse events in seven participants of the colchicine group versus 3 serious adverse events in two participants of the placebo group. Although there were numerically more side effects in the colchicine group, these were not felt to be related to the drug. In a follow-up assessment of the same study, Stamp and colleagues performed a multivariable analysis and found that the greatest risks of gout flares occurred in both those participants who had a gout flare in the month prior to starting allopurinol (odds ratio [OR] 2.65) and those who were started on 100 mg of allopurinol, rather than 50 mg (OR 3.21). Furthermore, when the prophylaxis was stopped at month 6, participants’ risks of developing another flare were higher among subjects in the colchicine group (OR 2.95), subjects who had at least one flare in the month prior to stopping study drug (OR 5.39), and subjects whose serum urate remained at greater than or equal to 0.36 mmol/L (6 mg/dL) (OR 2.85). Therefore, Stamp et al. felt that the duration of anti-inflammatory prophylaxis beyond 6 months would be of benefit for at least some individuals.

This study challenges the idea that colchicine prophylaxis should be started in every gout patient who is starting urate-lowering therapy. Specifically, prophylaxis may be most useful in patients who have a higher flare burden, particularly preceding the initiation of the allopurinol. However, this study did not look at other ULTs, such as febuxostat, and so cannot be generalized to all ULTs.

The optimal dosage of colchicine prophylaxis is also unclear. The ACR guidelines use the low dose of colchicine (0.6 mg) available in the US, while the EULAR guidelines mention 0.5–1 mg/day, to be adjusted according to renal function [4]. A recent study by Nieboer et al. appears to indicate that a lower dose of colchicine may be sufficient. The authors performed a retrospective cohort study in 808 patients at three rheumatology centers in the Netherlands starting xanthine oxidase inhibitors for gout along with colchicine prophylaxis. They found that 0.5 mg colchicine twice daily was not superior to 0.5 mg colchicine once daily at preventing gout flares, with an adjusted incidence rate ratio of 0.93 (CI 0.80–1.09) [5].

Another important consideration when managing gout patients with prophylaxis is the issue of concurrent medical illnesses. Gout is frequently comorbid with a variety of other conditions, particularly those that relate to metabolic syndrome, such as hypertension, hyperlipidemia, diabetes mellitus, and coronary artery disease [6,7]. Prophylactic agent choice is dependent upon patient tolerability and their comorbidities. For example, due to the frequency of chronic kidney disease (CKD) in gout patients, NSAIDs are often avoided in gout patients with moderate to severe CKD. Additionally, for those with the most severe type of kidney disease, colchicine is often used at lower doses or avoided entirely, but because of the failure of many clinical trials to include patients with advanced kidney disease, data on the effectiveness of these strategies are limited [8], and many of such patients are treated without prophylaxis or with prednisone, which can worsen edema in gout patients who suffer from severe CKD and exacerbate other comorbid conditions including heart failure.

Patients with gout are also at increased risk of myocardial infarctions. Cipolletta et al. have recently reported that MI risk is elevated immediately after an initial diagnosis of gout (up to 30 days) and after flares (up to 120 days) [9,10]. However, a subsequent study from the same group shows that colchicine use may lower this risk [11]. The authors performed a retrospective new-user cohort study from an English primary care database and included 99,800 patients with gout who initiated ULT. A total of 4063 patients had prior cardiovascular events. A total of 16,028 (16.1%) patients were given colchicine as prophylaxis alongside starting ULT. The authors then tracked the group for a period of 180 days after initiation of therapy and found that the colchicine group had a significantly lower risk of cardiovascular events, defined as fatal or non-fatal myocardial infarction or stroke, compared with the no-prophylaxis group, with a weighted hazard ratio of 0.82, manifested as 28.8 cardiovascular events per 1000 person-years in the prophylaxis group compared to 35.3 cardiovascular events per 1000 person-years in the treatment group.

This study further underlines the potential cardioprotective benefits of colchicine in gout patients [12,13] and may further help to stratify which patients should be started on prophylaxis upon initiation of ULT [14]. Recently published data have shown an increased risk of major adverse cardiovascular events when starting NSAIDs compared with colchicine or no prophylaxis, further supporting the idea that not all prophylaxis is equal [15]. Although no published data to date have considered the cardiovascular risks when starting prednisone for prophylaxis, analyses in the general population have shown that prednisone is also associated with increased cardiovascular and myocardial infarction risks [16–18].

Finally, the optimal length of prophylaxis is not clear. While 3–6 months is suggested by the guidelines, studies and meta-analyses have shown that about 29.7% of gout patients may flare in the three months after stopping prophylaxis, and 12.2% flare even further out [19]. These flares did not seem to be affected by prophylaxis duration, ULT class, or any other clearly measurable variables in those studies, making a clear recommendation for the termination of prophylaxis difficult. Additionally, in the original phase 3 study by Becker et al. comparing febuxostat and allopurinol, colchicine prophylaxis was discontinued after 8 weeks but the risk of flare did not return to pre-ULT baseline with either drug for approximately 6 months [20]. A case for longer prophylaxis with colchicine could be made for patients at higher cardiovascular risk or with a history of coronary artery disease, but further studies need to be conducted to evaluate the risks and benefits of such a strategy.

## 3. First-Line Agents for ULT

At present, the competitive xanthine oxidase inhibitor (XOI) allopurinol is the recommended first-line agent for all patients starting ULT [2], based on its efficacy, availability, safety, and affordability. However, real-world allopurinol effectiveness is much lower than its efficacy in clinical trials [21]. The allopurinol doses required to reach goal serum urate (i.e., <6 mg/dL) are, on average, above 300 mg [22], and can go as high as 800 mg in the US, or 900 mg in Europe. Many providers are unaware of this and routinely underdose their patients, thereby leading to higher than desirable urate levels and substandard care. While providers who apply treat-to-target protocols are more successful [23], most gout care occurs in primary care clinics, where providers are treating a variety of other conditions and are limited on time. Additionally, current American College of Physician gout treatment guidelines do not recommend a treat-to-target strategy, citing limited evidence and potentially dissuading primary care physicians from adopting a treat-to-target strategy. While a national US trial is underway testing the outcomes of ACR versus ACP guidelines, results are not expected for several years [24].

The use of allopurinol also entails consideration of the rare but serious risk of allopurinol hypersensitivity syndrome (AHS), necessitating stepwise titration of dose to avoid rapid overdosing. In gout patients with worsening CKD, titration is recommended to be further slowed. Another wrinkle in the use of allopurinol is the question of the necessity of testing for HLA-B*58:01 to reduce AHS risk. HLA-B*58:01 has been shown to be a significant risk factor for the development of AHS, and ACR guidelines conditionally recommend that HLA-B*58:01 be tested prior to initiation in high-risk populations, those of Southeast Asian descent, or African Americans [2]. However, the gene can be found in other populations (typically at a lower rate), and AHS has a very high morbidity and mortality. Better data are needed to determine which individuals should be tested, including cost–benefit analyses of various testing strategies. An additional prognostic model was recently published by Cipolletta et al., using primary care, hospitalization, and mortality data from the UK Clinical Practice Research Datalink primary care database, wherein adults residing in England newly prescribed allopurinol were followed for 100 days to assess whether a severe cutaneous adverse reaction was recorded in hospitalization and mortality records [25]. The authors found that age, chronic kidney disease stages 3, 4, and 5, an initial dose of 300 mg or higher of allopurinol, South Asian ethnicity, and other Asian ethnicity were the risks associated with 100-day allopurinol-induced severe cutaneous adverse events. This model can better help providers in screening patients at risk of allopurinol reaction and best tailor therapy to avoid allopurinol in individuals at the highest risk.

Optimal allopurinol titration is another increasingly studied approach to better gout management. Rheumatology guidelines recommend starting at low doses of urate-lowering therapy (≤100 mg of allopurinol, ≤40 mg of febuxostat etc.) And newer studies suggest that a “start low, go slow” approach, using 50 mg allopurinol and titrating in 50 mg increments, may be best for preventing flares and side effects from titration [2,26].

Febuxostat, a second-line agent that non-competitively inhibits xanthine oxidase, has a simpler titration schedule than allopurinol, ranging only from 40 mg to 80 mg (and up to 120 mg in countries outside the United States). In head-to-head comparisons, febuxostat works equally well as allopurinol at lowering serum urate to the goal, but often with fewer titration steps and less need to reduce initial doses and titration dosing in patients with kidney disease [27]. Additionally, there is no risk of AHS or need to check the HLA-B*58:01 gene in at-risk populations, as there is with allopurinol initiation. There are some data suggesting that the risk of an allergic skin reaction is slightly increased in patients switched to febuxostat after having a cutaneous reaction to allopurinol, with Bardin et al. finding a 9.1% risk of skin reaction in patients switched to febuxostat after a skin reaction to allopurinol, compared with only 2.5% of patients without a skin reaction to allopurinol developing a skin reaction to febuxostat [28].

While febuxostat’s cost per pill is higher than that of allopurinol, and although initial studies showed that allopurinol was a cheaper option than febuxostat, febuxostat appears to be more cost-effective due to increased treatment success [29,30].

Febuxostat has been limited in its use due to a black box warning for cardiovascular mortality based on the results of the CARES trial [31]. The CARES trial had some important flaws, including a lack of placebo control and a high withdrawal rate of subjects in both groups. Therefore, several follow-up studies have been conducted, including the FAST trial [32], a similar trial in Europe which evaluated a population at a similarly higher cardiovascular risk, but had much fewer lost to follow-up and better accounted for patients being on appropriate cardiac therapy. The authors of the FAST trial found no increased risk of adverse cardiovascular outcomes in the febuxostat compared with allopurinol arm. Multiple other studies have since shown a similar lack of increased risk in patients taking febuxostat compared with allopurinol, including a Taiwanese nationwide study [33]. Despite this evidence, the black box warning in the US remains, creating an area of concern for many providers, particularly given the high rates of cardiovascular disease in gout patients at baseline.

When a patient with gout requires febuxostat and has a history of cardiovascular disease, we will often discuss the risks and benefits of starting febuxostat with the patient and their cardiologist, and, if they are on a stable regimen for their cardiovascular disease and have had no recent myocardial infraction, we feel comfortable starting febuxostat in that patient.

## 4. Second-Line Agents for ULT

The only other oral ULT agent currently approved in the US is probenecid, an inhibitor of renal transporters of urate in the kidneys, increasing uric acid excretion to lower serum urate. However, probenecid is not effective at GFRs below 50, requires BID dosing, and has a relatively high rate of causing kidney stone formation, thereby limiting its use predominantly as a second line on top of XOIs.

Benzbromarone, another uricosuric agent, is registered in 20 countries throughout Asia, South America, Europe, and New Zealand and has been widely withdrawn from many markets due to potential hepatotoxicity risks [34]. However, newer data indicate that the risks of liver disease may have been overestimated, with several studies showing that it is as effective as allopurinol and febuxostat and potentially less hepatotoxic [35,36]. Therefore, in those countries where it is available, it should be considered a safe option for ULT in gout patients, provided there is proper monitoring of liver function. Lesinurad, another uricosuric agent, has been completely withdrawn from production. Therefore, neither of these agents, despite improved urate-lowering properties compared with probenecid, are available for most providers. Newer agents targeting renal uric acid secretion or reabsorption are currently in phase 2 and 3 trials and may alter the treatment landscape going forward.

The final approved agent, pegloticase, a pegylated uricase, is given as an infusion every 2 weeks. Pegloticase is extremely effective at urate lowering, but relatively high rates of anti-drug antibody generation and potential for infusion reactions require concomitant immunosuppression with methotrexate and pre-infusion steroids, making the agent more complex to dose and discouraging inexperienced providers [37].

A newer uricase, SEL-212, which combines the uricase pegadricase with a rapamycin-containing nanoparticle ImmTOR^™^ (Selecta Biosciences, Watertown, MA, USA) is also being studied as an alternative to pegloticase. It benefits from the combination with ImmTOR acting to decrease the risk of loss of tolerance due to anti-drug antibodies, thereby allowing for twice-monthly infusions without the need for an oral immunosuppressive agent. When compared with pegloticase, SEL-212 was found to be comparable in efficacy and tolerability with pegloticase without the need for oral immunosuppression [38].

Dotinurad, a novel selective urate reabsorption inhibitor which is currently approved in Japan and being studied in other countries, may help to further increase urate-lowering options, as it appears as effective or more effective than currently approved XOIs, as well as effective and tolerable in patients with CKD [39,40].

A new XOI, tigulixostat [41], which may help broaden the choices in the future is also currently in trials.

## 5. Medications with Secondary Urate-Lowering Effects

A variety of medications primarily used for other medical conditions may also be used to lower serum urate. For example, losartan, a commonly used angiotensin receptor blocker for hypertension, was found to significantly lower serum urate by 0.23 mg/dL when compared to the DASH diet in hypertensive participants over 8 weeks, though the subjects were normouricemic, with mean serum urate of 5.2, and did not have gout [42]. More recently, data have shown the potentially robust effects of urate lowering of sodium-glucose cotransporter type 2 inhibitors (SGLT2-is), used for diabetes mellitus, in lowering serum urate by over 1 mg/dL, when compared to sulfonylurea in gout patients [43].

While these medications are not approved for gout, their off-label use, particularly in patients suffering from hypertension and/or diabetes, both of which are common comorbidities of gout [6], appears to be increasing. Additional studies would help to elucidate whether and when these agents may be viable treatments and/or adjuvant therapies for gout management.

## 6. Debulking Therapy in Tophaceous Gout

Among the most challenging gout patients are the significant minority with tophaceous deposition. These patients often have significant pain and limitations in their activities of daily living due to their disease [44]. However, recommendations for initial management of these patients are largely the same as those for patients with less severe disease, including the initiation of XOIs. The initial use of uricases for debulking therapy, followed by oral agents for maintenance, may prove an effective treatment option. Uricases rapidly lower serum urate and have been shown to rapidly shrink tophi [45,46]. Despite their cost and risk of adverse reactions due to immunogenicity, an initial short course of uricase therapy in tophaceous patients may be a good option in the long term, allowing gout patients with tophaceous deposition to have more rapid control of their debilitating disease [47].

## 7. Optimal Serum Urate Goal

The current serum urate goal when starting most patients with gout on ULT is <6.0 mg/dL based on current ACR guidelines. However, in many gout patients on ULT, there remains evidence of MSU deposition even after 12 months at target serum urate concentrations [48]. Interestingly, prior ACR guidelines and EULAR guidelines have recommended considering a target ≤ 5.0 mg/dL for individuals with more severe gout symptoms, such as frequent flares or tophi [2,4]. Such a strategy is supported by data from Perez-Ruiz et al. and from Kelly et al., indicating that lower urate targets may promote more rapid MSU crystal dissolution and tophus resolution [48,49]. Indeed, Kelly and colleagues found that the most rapid dissolution of MSU crystals was seen at serum urates below 0.24 mmol/L (<4 mg/dL), further supporting the idea that for gout patients with greater MSU deposition, more aggressive urate lowering, at least initially, may be more effective in more rapidly resolving recurrent flares and shrinking tophi.

## 8. Conclusions

While there are well developed treatment recommendations in managing gout and lowering serum urate, several important questions remain if we are to achieve improvements in our ability to diagnose and recognize MSU deposition. Such improvements may allow for more specifically tailored advice for different gout patients, depending on the severity of their gout.

Given the frequency of comorbidities among gout patients, including cardiovascular disease, hypertension, and diabetes, prescribing medications that treat both gout and these conditions concomitantly may lower medication burden, increase patient adherence to medications, and improve patients’ health outcomes.

The growing number of ULT options also helps broaden physicians’ armamentarium in lowering serum urate and may allow patients who have had multiple adverse reactions to existing therapies to be better managed.

The future of urate-lowering therapy in gout is bright, as we continue to understand more about this disease, and new research in the genetics of gout may lead to a future where we are able to cure gout and correct the imbalance leading to hyperuricemia and crystal formation.

## Data Availability

As this was a review article, there is no original data used to write this manuscript, and all data has been cited within the reference section.
